# Transcriptomic analysis of sweet potato under dehydration stress identifies candidate genes for drought tolerance

**DOI:** 10.1002/pld3.92

**Published:** 2018-10-30

**Authors:** Kin H. Lau, María del Rosario Herrera, Emily Crisovan, Shan Wu, Zhangjun Fei, Muhammad Awais Khan, Carol Robin Buell, Dorcus C. Gemenet

**Affiliations:** ^1^ Department of Plant Biology Michigan State University East Lansing Michigan; ^2^ International Potato Center Lima Peru; ^3^ Boyce Thompson Institute Cornell University Ithaca New York; ^4^ Plant Resilience Institute Michigan State University East Lansing Michigan; ^5^ Present address: Plant Pathology and Plant‐Microbe Biology Section Cornell University Geneva New York

**Keywords:** drought, expression, *Ipomoea batatas*, polyethylene glycol, RNA‐Seq, sweet potato

## Abstract

Sweet potato (*Ipomoea batatas* [L.] Lam.) is an important subsistence crop in Sub‐Saharan Africa, yet as for many crops, yield can be severely impacted by drought stress. Understanding the genetic mechanisms that control drought tolerance can facilitate the development of drought‐tolerant sweet potato cultivars. Here, we report an expression profiling study using the US‐bred cultivar, Beauregard, and a Ugandan landrace, Tanzania, treated with polyethylene glycol (PEG) to simulate drought and sampled at 24 and 48 hr after stress. At each time‐point, between 4,000 to 6,000 genes in leaf tissue were differentially expressed in each cultivar. Approximately half of these differentially expressed genes were common between the two cultivars and were enriched for Gene Ontology terms associated with drought response. Three hundred orthologs of drought tolerance genes reported in model species were identified in the *Ipomoea trifida* reference genome, of which 122 were differentially expressed under at least one experimental condition, constituting a list of drought tolerance candidate genes. A subset of genes was differentially regulated between Beauregard and Tanzania, representing genotype‐specific responses to drought stress. The data analyzed and reported here provide a resource for geneticists and breeders toward identifying and utilizing drought tolerance genes in sweet potato.

## 1 INTRODUCTION

Sweet potato (*Ipomoea batatas* [L.] Lam.) is grown worldwide for its nutrient‐rich storage roots. In Sub‐Saharan African (SSA), sweet potato is a subsistence crop for small holder farmers and is a major source of energy and nutrition; as a consequence, it is an important food security crop (Low, Mwanga, Andrade, Carey, & Ball, 2017; Low et al., 2009; Mwanga & Ssemakula, 2011). Drought is a major concern for agriculture in many regions and is a particularly important issue among poor smallholder farmers who often lack the capacity to irrigate their crops, including sweet potato (Low et al., 2009). Screens for drought tolerance among sweet potato clones that grow well in SSA environments such as Mozambique (Maquia et al., 2013) and Kenya (Kivuva, Githiri, Yencho, & Sibiya, 2015) have shown that drought tolerance varies greatly in sweet potato germplasm, with the relative performance of sweet potato varieties in terms of yield shifting substantially between drought and non‐drought conditions. The presence of genetic variation for drought tolerance among sweet potato germplasm suggests that drought tolerance loci can be introgressed into drought‐sensitive cultivars. Thus, understanding the genetic and molecular basis of drought tolerance will aid in breeding for high‐performing, drought tolerant sweet potato cultivars.

Drought stress induces a set of physiological responses with stomatal closure to reduce water loss through transpiration being the most immediate and important (reviewed in Osakabe, Osakabe, Shinozaki, & Tran, 2014). At the onset of drought, abscisic acid (ABA) levels are elevated, initiating signaling pathways that modulate guard cell osmolarity to achieve stomatal closure. As a result, components of the ABA signaling pathway as well as guard cell ion pumps are essential for proper stomatal closure during drought, leading to drought tolerance. However, control of stomatal closure during drought involves compromising between limiting water loss and maintaining CO_2_ uptake for photosynthesis, which ultimately impacts crop yield. Thus, understanding the intricate processes that modulate drought responses such as stomatal closure and resiliency of photosynthetic rates during drought are crucial aspects of optimizing drought tolerance in crop plants.

Beauregard is a US‐bred variety with low dry matter and orange flesh due to high levels of carotenoids (Rolston et al., 1987), while Tanzania is a popular Ugandan landrace with high dry matter and cream flesh (Mwanga et al., 2001). Though a Tanzania × Beauregard population (Cervantes‐Flores, Yencho, Kriegner, et al., 2008) has been used to map quantitative trait loci (QTL) for root‐knot nematode resistance, dry matter, starch, and β‐carotene content (Cervantes‐Flores, Yencho, Pecota, Sosinski, & Mwanga, 2008; Cervantes‐Flores et al., 2011), drought tolerance traits have not yet been investigated in this population. However, we have an ongoing project to map QTL for drought‐related traits using a population derived from the reciprocal cross, Beauregard × Tanzania. Promisingly, Kivuva et al. (2015) in a study involving 84 total genotypes showed that Beauregard differs from Tanzania in several drought response traits. Greenhouse‐grown plantlets derived from cuttings of Tanzania stay green longer under drought conditions than those from Beauregard by 6–7 days, suggesting greater drought tolerance in Tanzania (Supporting Information Figure S1a). Mature plants grown at two different sites in Kenya under irrigated and rain‐fed conditions resulted in a larger decrease in chlorophyll content in Tanzania compared to Beauregard, yet the number of storage roots and the fresh storage root weight were more sensitive in Beauregard than in Tanzania (Supporting Information Figure S1b–d).

A few studies have examined global transcriptomic responses for drought‐related traits in different *Ipomoea* species. Yang et al. (2018) recently reported a three‐way combined *de novo* transcriptome for whole plants of PEG‐treated *Ipomoea triloba*,* I. batatas* (cv. Kokei No. 14) and a somatic hybrid between the two species, finding genes that are differentially regulated in the *I. batatas* parent compared to either *I. triloba* or the hybrid, which are more drought tolerant. Other de novo transcriptome approaches have been reported for hexaploid *Ipomoea trifida* and *Ipomoea imperati* for drought and salt stress, respectively (Peng et al., 2017; Solis, Baisakh, Brandt, Villordon, & Bonte, 2016). In this study, global gene expression patterns in leaf tissue of two varieties of *I. batatas*, Beauregard, and Tanzania, during PEG‐simulated drought stress were characterized with RNA‐Sequencing (RNA‐Seq); diploid *I. trifida*, which is closely related to *I. batatas* and is a putative progenitor species (Austin, 1988; Kobayashi, 1983), was used as the reference genome sequence (http://sweetpotato.plantbiology.msu.edu/gt4sp_download.shtml). Differentially expressed genes (DEGs) were identified, revealing candidate genes for improving drought tolerance in sweet potato. In addition, genes with unique expression patterns in the two cultivars were classified into co‐expression clusters showing enrichment for biological processes that could contribute to phenotypic differences in their drought responses. Data from this study will be useful in identifying candidate genes within drought tolerance QTL and improving drought tolerance in this key food security crop.

## 2 MATERIALS AND METHODS

### 2.1 Determination of optimal conditions to induce dehydration stress

In vitro plants of Beauregard (CIP440132) and Tanzania (CIP440166) were obtained from the International Potato Center (CIP) Genebank and nodes were cultured to form plantlets on MPB medium (Murashige and Skoog salts, 3% sucrose, 2 mg/L calcium pantothenate, 100 mg/L l‐arginine, 200 mg/L ascorbic acid, 20 mg/L putrescine‐HCl, 10 mg/L GA_3_, 0.3% Phytagel, pH 5.7, autoclaved at 121°C for 15 min as recommended by the supplier) in culture tubes (three per tube). Shoot cultures were kept in a growth room at 27 ± 2°C, 3,000 lx, and 12 hr light/12 hr dark.

Polyethylene glycol (PEG 6000, Sigma) concentrations were chosen to create osmotic pressure levels between field capacity (−0.033 mpa) and permanent wilting (−1.5 mpa), as estimated based on osmotic pressure values reported for different PEG 6000 concentrations (http://www.plantstress.com/methods/peg.htm; Michel & Kaufmann, 1973). After 21 days of growth in solid MPB media, the plants were transferred to 35 ml of 15%, 20%, and 25% PEG6000 liquid MPB media, representing osmotic pressure values of −0.295, −0.491, and −0.735 mpa, respectively. Three replicates were used. Cultures were maintained in a growth chamber under the conditions described above and stress symptoms were evaluated at 4, 24, 48 hr, and 21 days after stress application.

### 2.2 Plant growth conditions for RNA‐Seq

For both varieties, one node was cut from the donor plant and placed in a 16 × 125 mm tube containing solid MPB media. Only the internode was submerged into the solid media to allow the node to grow. Fourteen days after planting, six plantlets with similar size were selected and transferred to another vessel containing liquid MPB media. A thin plate of acrylic plastic containing small holes was used to support plantlets and to allow leaf expansion. Three biological replicates and three controls were used. Cultures were maintained in the growth chamber under the same conditions as described above. At 21 days after planting, the initial liquid media was removed and replaced with liquid media containing 25% PEG in liquid MPB medium. Leaf samples were collected for RNA extraction at 24 and 48 hr after stress (HAS) imposition, flash frozen, and stored at −80°C. RNA extraction was performed with 200 mg of leaf tissue for each sample using TRIzol (Invitrogen) according to supplier instructions.

### 2.3 Library construction, sequencing, and expression estimation

Strand‐specific RNA‐Seq libraries (one library for each of three biological replicates per experimental condition; Supporting Information Table S1) were constructed using the protocol described in Zhong et al. (2011) and sequenced on an Illumina HiSeq 2500 platform. Reads were trimmed using CutAdapt (v1.8.3; Martin, 2011), removing up to two adapter sequences from each read, retaining only reads with a minimum read length of 31 nt and trimming the 3′ end with a quality cut‐off of 10. Reads were trimmed to 100 nt using FASTX (v0.0.14; http://hannonlab.cshl.edu/fastx_toolkit/) as a subset of libraries were sequenced to a read length of 151 nt instead of 101 nt. Reads flagged by the Illumina pipeline as low quality with “Y” were removed. Mononucleotide repeats of T and A 10‐mers were removed up to 10 times using CutAdapt (v1.8.3), retaining only reads with a minimum read length of 31 nt. Libraries were run on multiple lanes (technical replicates) and were mapped independently to the *I. trifida* genome assembly v3 (http://sweetpotato.plantbiology.msu.edu/gt4sp_download.shtml) using TopHat2 (v2.1.1; Kim et al., 2013) implementing Bowtie2 (v2.2.9; Langmead & Salzberg, 2012) with the options: –min‐intron‐length 10, –max‐intron‐length 5000, –max‐multihits 20, –library‐type fr‐firststrand, –min‐segment‐intron 10, –max‐segment‐intron 5000, –b2‐N 1, –read‐mismatches 3, and –read‐edit‐dist 3.

Cufflinks (v2.2.1; Trapnell et al., 2010) was used to calculate fragments per kilobase exon model per million mapped reads (FPKMs) for the set of high‐confidence *I. trifida* gene models (v3) using the options: –multi‐read‐correct, –min‐intron‐length 10, –max‐intron‐length 5000, –library‐type fr‐first strand. One technical replicate was removed because of a markedly lower alignment rate compared to other technical replicates. Transcripts *itf01g31740.t1*,* itf02g16070.t2*,* itf03g01940.t1* were removed because these had identical coordinates as another isoform of the same gene. Correlations between different sequencing runs of the same library were quantified using Pearson Correlation Coefficients from FPKMs in R (pairwise.complete.obs, method = pearson). Technical replicates were highly correlated, with most correlation coefficients above 0.95 and others above 0.90. The alignment files for technical replicates were merged using the MergeSamFiles tool in PicardTools (v2.1.1; https://broadinstitute.github.io/picard/), and used for calculating FPKMs for each biological replicate (Supporting Information Table S2) and for differential expression analysis.

### 2.4 Identification of differentially expressed genes

Gene annotations (v3; http://sweetpotato.plantbiology.msu.edu/gt4sp_download.shtml) were converted from GFF3 to GTF format using the “gffread” script included with Cufflinks (v2.2.2). Uniquely mapping reads overlapping gene models were counted using htseq‐count in HTSeq (v0.6.1p1; Anders, Pyl, & Huber, 2015) with the options: –stranded = reverse –minaqual = 10 –type = exon –mode = union.

Read counts were used to detect differential expression with DESeq2 v1.16.1 (Love, Huber, & Anders, 2014). For differential expression between control and PEG treatment at each time‐point in each variety, unique combinations of treatment, time‐points, and variety were treated as a single factor, and the contrasts function in DESeq2 was used to test whether log2 fold change was equal to 0 for each pair of control and PEG treatment levels. An adjusted *p*‐value cut‐off of 0.05 and a log2 fold‐change threshold of 1.5 were used. The “lfcShrink” function was used to restrain high log2 fold changes for genes with low expression levels.

To detect genes that responded differentially to PEG between Beauregard and Tanzania at either time‐point or both time‐points, the full model, ~treatment + variety + time_point + variety:time_point + treatment:time_pt + treatment:variety:time_point, was tested against a reduced model of, ~treatment + variety + time_point + variety:time_point + treatment:time_pt using a likelihood ratio test implemented in DESeq2 and an adjusted *p*‐value cut‐off of 0.01.For K‐means clustering, log2 fold changes calculated by DESeq2 were obtained for significant genes and mean‐centered by subtracting the arithmetic mean for each gene. K‐means clustering was conducted using the “kmeans” function in R with the settings, centers = 16, nstart = 25, iter.max = 100, algorithm = “MacQueen” (MacQueen, 1967). A K of 16 was chosen because relatively little reduction in total within‐cluster variation was observed for larger values.

### 2.5 Gene ontology term enrichment analysis

Groups of DEGs or clusters with similar expression patterns were tested for enriched gene ontology (GO) terms using the “weight01” algorithm and Fisher's exact test implemented in topGO v2.28.0 (Alexa & Rahnenfuhrer, 2016) using all genes with GO terms as the background. *p*‐values were corrected for multiple testing using the “p.adjust” function in R to implement the FDR method (Benjamini & Hochberg, 1995), and filtered at the 0.05 level.

### 2.6 Orthology analysis

OrthoFinder v1.1.3 (Emms & Kelly, 2014) was run with the longest peptide isoforms from the predicted proteomes of *Amborella trichopoda* v1.0, *Arabidopsis thaliana* TAIR10, *I. trifida* v3, *Oryza sativa* v7, and *Solanum lycopersicum* iTAG2.4, obtained from Phytozome v12.1.5 (Goodstein et al., 2012).

## 3 RESULTS AND DISCUSSION

### 3.1 Establishing effective dehydration conditions

**Figure 1:**
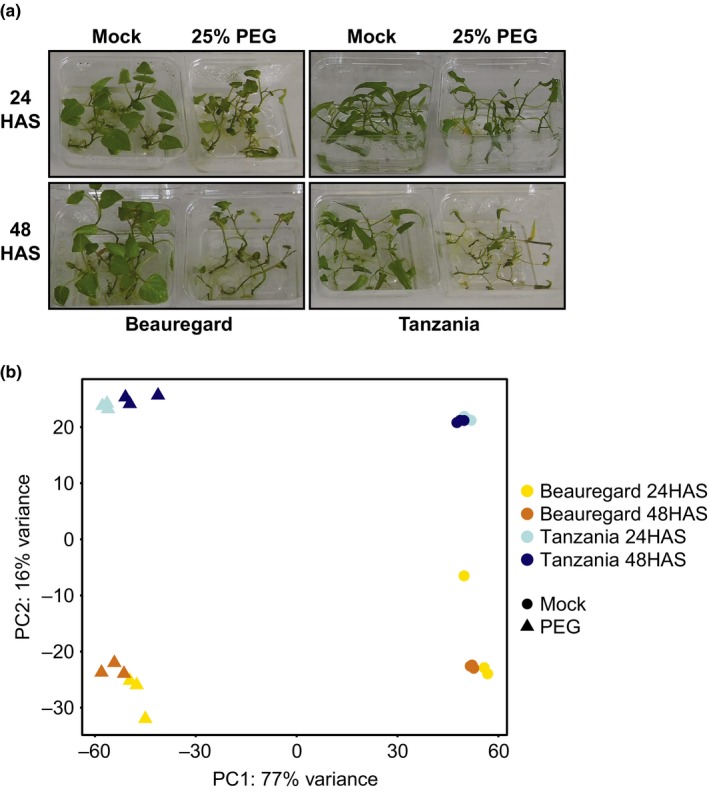
Phenotypic and genetic response of Beauregard and Tanzania sweetpotato varieties to the application of polyethylene glycol (PEG). (a) Plantlets incubated in untreated media or 25% PEG media at 24 and 48 hr after stress (HAS). (b) Principal component analysis of individual biological replicates using variance stabilizing transformed read counts. Each point represents a biological replicate

Drought conditions were simulated by adding PEG to the media that plantlets were grown in. No stress symptoms were observed up to 6 HAS. However, wilted and chlorotic leaves were observed in PEG‐treated plantlets of both varieties by 24 HAS, and the severity increased at 48 HAS (Figure 1a), with plants dying after 21 days. In contrast, leaves on plantlets not treated with PEG remained green and turgid. No visible differences between the stress phenotypes of Beauregard and Tanzania were observed at these plantlet stages. An osmotic pressure of −0.735 mpa (25% PEG 6000) was chosen for the actual stress experiment for RNA‐Seq analysis as it elicited strong differences between treated and untreated plants.

### 3.2 Global gene expression profiles are conserved between Beauregard and Tanzania

**Figure 2:**
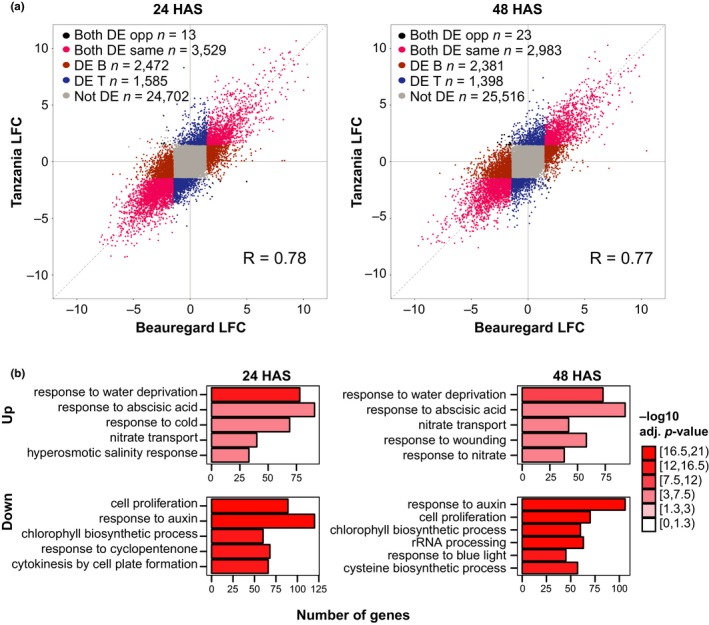
Conserved expression responses to polyethylene glycol in Beauregard and Tanzania. (a) Log2 fold change (LFC) of gene expression abundances in Beauregard and Tanzania at 24 and 48 hr after stress (HAS). “DE B” and “DE T” indicate genes differentially expressed in only Beauregard or Tanzania, respectively. (b) The most significantly enriched biological process gene ontology terms among genes differentially expressed in both Beauregard and Tanzania at 24 and 48 HAS

To assess the quality of the biological replicates and the extent of gene expression variation explained by the experimental variables, a principal component analysis (PCA) was conducted (Figure 1b). Tight clustering of biological replicates indicated that the majority of the gene expression variation observed was due to the experimental conditions. Greater than 75% of variation was explained by the first principal component, which corresponded predominantly to the addition of PEG. In comparison, varietal effects of Beauregard and Tanzania, and the time after PEG application contributed to a much smaller portion of the expression variation. In support of the PCA results, expression fold changes of mock treatment vs. PEG treatment were significantly correlated between Beauregard and Tanzania at both 24 and 48 HAS (*p* < 2.2e‐16 for both; Figure 2a). Indeed, genes with the largest fold change between mock and PEG treatments were differentially expressed in the same direction and at similar magnitude in Beauregard and Tanzania (Figure 2a). Conversely, a large number of genes with less extreme fold changes were differentially expressed in one variety but not the other, indicating that a substantial portion of dehydration‐responsive genes are unique to each genotype.

Considering that Beauregard is one of the predominant cultivars for large, commercial‐scale cultivation in the U.S. while Tanzania is a landrace commonly grown by smallholder farmers in Uganda and other East African countries (Carpena, 2009), these two varieties are likely adapted to different environmental conditions, and shared genetic responses to PEG may be involved in critical drought tolerance or survival mechanisms. At 24 HAS, 2,216 and 1,809 genes were upregulated while 3,798 and 3,318 genes were downregulated in Beauregard and Tanzania, respectively. At 48 HAS, numbers of upregulated genes were comparable to 24 HAS, but downregulated genes decreased to 3,285 and 2,482. Among genes differentially expressed in the same direction in both genotypes, similar GO terms were enriched at 24 and 48 HAS. Consistent with a drought response, “water deprivation,” “response to ABA,” and “nitrate transport” were among the top biological processes (Figure 2b). Enrichment for the “response to ABA” GO term is expected because drought utilizes ABA signaling to induce stomatal closure in order to reduce water loss via transpiration (reviewed in Osakabe et al., 2014). An important part of this ABA signaling pathway is the transport of ABA to stomatal guard cells by ABA transporter proteins, such as NITRATE TRANSPORTER 1.2 (NRT1.2; Kanno et al., 2012), explaining the “nitrate transport” GO term enrichment. Indeed, upregulated genes enriched for the nitrate transport GO term included *NRT1.2* homologs, *NRT1.7* and – at 48 HAS only – *NRT1.5*, and an unrelated nitrate transporter, *NRT2.7* (Dechorgnat et al., 2011; Supporting Information Dataset S1). Alternatively, the “nitrate transport” GO term may be involved in the accumulation of free amino acids such as proline that are hypothesized to help maintain cell turgor by decreasing water potential inside cells (Stewart & Lee, 1974). Upregulated genes enriched for the “water deprivation” GO term (Supporting Information Dataset S1) included well‐known drought tolerance genes such as *DEHYDRATION‐RESPONSIVE ELEMENT‐BINDING PROTEIN 2A* (*DREB2A*) which encodes a drought‐responsive transcription factor (Liu et al., 1998; Sakuma et al., 2006) and *LATE EMBRYOGENESIS ABUNDANT* (*LEA*), a family of stress‐inducible genes that encode proteins that have been implicated in diverse drought tolerance mechanisms such as preventing protein aggregation and membrane protection during dehydration (Goyal, Walton, & Tunnacliffe, 2005; Tolleter et al., 2007).

For downregulated genes, the most significant GO terms were primarily involved in cell division, response to auxin and chlorophyll biosynthesis (Figure 2b). Stomatal closure leads to reduced flux through the Calvin cycle, limiting the supply of NADP^+^ electron acceptors to the electron transport chain in the chloroplasts causing an overaccumulation of electrons (Noctor, Mhamdi, & Foyer, 2014; Osmond & Grace, 1995). Thus, downregulation of genes involved in chlorophyll biosynthesis may represent an effort to avoid overaccumulation of electrons by reducing photosynthetic capacity. Regardless, reduced chlorophyll content due to drought has been reported in multiple plant species (Jiang & Huang, 2001; Li, Pezeshki, & Goodwin, 2004; Sarker, Rahman, & Paul, 1999) and as a consequence of decreased photosynthesis, less resources are available to support tissue growth thereby explaining the downregulated GO terms of “cell proliferation” and the “response to auxin,” an important regulator of cell and tissue growth. Leaves, the tissue that RNA was extracted from for this study, are often more susceptible to reduced growth rate compared to roots and it is hypothesized that reduced leaf area helps limit water loss due to transpiration (Aguirrezabal et al., 2006; Pace, Cralle, El‐halawany, Cothren, & Senseman, 1999; Xu et al., 2015). These overall expression patterns are consistent with a drought response and indicate that dehydration stress was successfully imposed by the PEG treatment.

### 3.3 A group of leucine‐rich kinases are downregulated at 24 HAS

**Figure 3:**
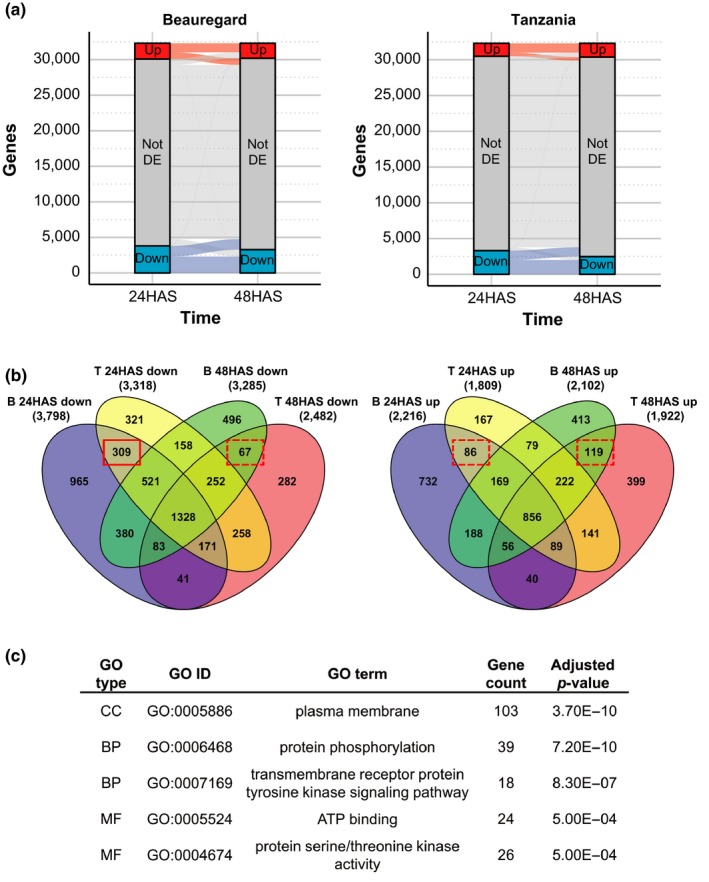
Time‐point specific differentially expressed genes (a) Differentially expressed genes at 24 and 48 hr after stress (HAS) in Beauregard and Tanzania. Genes labeled “up” or “down” were significantly upregulated or downregulated, respectively. (b) Overlap between genes significantly downregulated or upregulated in each variety (B: Beauregard, T: Tanzania) and at each time‐point. Red rectangles indicate genes tested for GO term enrichment and dashed outlines indicate weak or insignificant enrichment. (c) The most significant GO terms for genes downregulated in both Beauregard and Tanzania at 24 HAS but not 48 HAS (solid red rectangle in panel b)

For both directions of regulation, hundreds of DEGs were unique to a particular time‐point in each variety, indicating that dynamic regulatory changes take place between 24 HAS and 48 HAS (Figure 3a,b). To examine their biological significance, GO term enrichment was tested for the DEGs that were specific to a particular time‐point and common to both varieties (Figure 3b). While only weakly significant GO terms were observed for the other three classes of time‐specific DEGs, genes downregulated at 24 HAS only were strongly enriched for protein phosphorylation and plasma membrane GO terms (Figure 3c). Out of the 39 genes downregulated at only 24 HAS that had a protein phosphorylation GO term, 24 were annotated as leucine‐rich repeat protein kinases (LRR‐PK) or leucine‐rich receptor‐like kinases (LRR‐RLK) and five were other types of kinases (Supporting Information Dataset S2). The involvement of these genes in PEG response in sweet potato is consistent with previous transcriptomic analyses that have shown many LRR‐RLK genes in Arabidopsis to be responsive (both down and upregulated) to osmotic stress (Chae, Sudat, Dudoit, Zhu, & Luan, 2009; Lehti‐Shiu, Zou, Hanada, & Shiu, 2009).

### 3.4 Identification and analysis of orthologs of drought tolerance candidate genes

**Figure 4:**
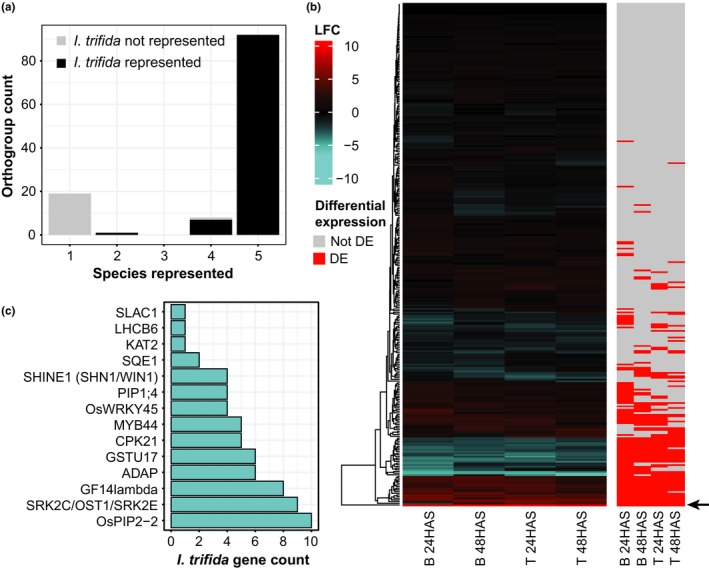
*Ipomoea trifida* orthologs of drought tolerance candidate genes identified in Arabidopsis, rice, and tomato. (a) Species representation of orthogroups containing DroughtDB genes. (b) Log2 fold changes (left; polyethylene glycol/control) and differential expression state (right; grey: not differentially expressed; red: differentially expressed). The arrow indicates itf03g07310 (*TAS14*). (c) Counts of *I. trifida* genes in orthogroups containing drought tolerance candidate genes significantly downregulated in all four variety‐time point combinations

DroughtDB (Alter et al., 2015) is a manually curated database of ~200 drought tolerance candidate genes compiled mainly from knockout, knockdown, and overexpression experiments in 38 different plant systems. Orthogroups were identified for the predicted proteomes of *I. trifida*,* A. trichopoda* (representing the most basal clade among angiosperms) and three highly represented species in DroughtDB, *A. thaliana*,* O. sativa,* and *S. lycopersicum*. Eighty‐three percent of orthogroups containing candidate drought tolerance genes were conserved by at least four species, consistent with important biological roles for these genes (Figure 4a). In total, there were 300 DroughtDB orthologs in *I. trifida*, and 122 of these were differentially expressed in at least one variety‐time point combination (Figure 4b).

#### 3.4.1 Upregulated candidate genes include a LEA gene and ABA metabolism genes

We focused on the 21 upregulated (*itf12g21010* was downregulated at 24 HAS in Beauregard) and 16 downregulated drought candidate genes that were differentially expressed at all four variety‐time point combinations, reasoning that these genes are strong candidates to be involved in drought stress responses (Figure 4b; Supporting Information Figure S2). A *TAS14* homolog (*itf03g07310*) was upregulated by >8 log2 fold change across all four variety‐time points combinations, substantially higher than any other drought tolerance candidate gene (Figure 4b). TAS14 is a LATE EMBRYOGENESIS ABUNDANT (LEA) protein of the dehydrin subfamily and its expression is induced by salt, ABA or mannitol treatment in tomato (Godoy, Pardo, & Pintor‐Toro, 1990). Overexpression of *TAS14* in tomato under drought stress increases fruit biomass several fold compared to wild‐type plants while producing no discernable deleterious effects under unstressed conditions (Muñoz‐Mayor et al., 2012). The mechanism of TAS14 action may be related to an increase in soluble sugars in the leaves that coincides with higher water potential and cell turgor in drought‐stressed plants over‐expressing *TAS14* (Muñoz‐Mayor et al., 2012). Other candidate genes upregulated in all four experimental conditions include the drought‐inducible DREB2A transcription factor (Liu et al., 1998) and several components of the ABA signaling pathway. These include the ABA and drought‐inducible bZIP transcription factor ABF3/ABF4/AREB1/OsbZIP23 that activates genes with ABRE (ABA responsive element) promoter elements (Kang, Choi, Im, & Kim, 2002; Uno et al., 2000; Xiang, Tang, Du, Ye, & Xiong, 2008), and the ATP‐binding cassette (ABC) ABA transporters, ABCG22, 25 and 40 (Kang et al., 2010; Kuromori, Sugimoto, & Shinozaki, 2011; Kuromori et al., 2010). Besides the transport of ABA, its metabolism was also implicated in dehydration response in sweet potato. *NCED* encodes 9‐cis‐epoxycarotenoid dioxygenase, an ABA biosynthesis enzyme whose induction correlates with increases in ABA during drought stress (Iuchi et al., 2001). Conversely, CYP707A1 and 3 are ABA catabolic enzymes that are induced by both dehydration and rehydration in Arabidopsis, allowing controlled ABA responses and rapid disengagement of ABA responses, respectively (Kushiro et al., 2004; Saito et al., 2004; Umezawa et al., 2006).

#### 3.4.2 LHCSB6 and SLAC1 are unexpectedly downregulated during water stress

Surprisingly, some drought tolerance candidate genes that improve drought tolerance when overexpressed or functional in other species were downregulated in both varieties at both time‐points. *SLOW ANION CHANNEL‐ASSOCIATED 1* (*SLAC1*; Negi et al., 2008; Vahisalu et al., 2008) and *LIGHT‐HARVESTING CHLOROPHYLL A/B‐BINDING6* (*LHCB6*; Jansson, 1999) are two such genes and, intriguingly, these two genes lack paralogs in *I. trifida*, suggesting that their down‐regulation during dehydration stress cannot be compensated by the activity of other genes (Figure 4c). KAT2 was another gene downregulated at all four variety‐time point combinations that has no paralogs, but the downregulation of this gene, encoding an inwardly rectifying K^+^ channel*,* is consistent with the need for net K^+^ efflux from guard cells during stomatal closure (Schroeder, Hedrich, & Fernandez, 1984).

In contrast, the downregulation of *LHCB6* and *SLAC1* may have detrimental effects on stomatal closure during drought, and restoring their functions by overexpression may improve drought tolerance in sweet potato. *LHCSB6* encodes a protein associated with photosystem II (Jansson, 1999) that, in Arabidopsis, inhibits ABA‐mediated stomatal closure when expression is knocked down and enhances ABA‐mediated stomatal closure when overexpressed (Xu et al., 2012). Due to its role in mediating stomatal closure, the knockdown mutant of *LHCSB6* has increased drought sensitivity, with visibly more severe reductions in leaf size and greenness compared to wildtype (Xu et al., 2012). SLAC1 is an anion efflux channel protein that is essential for proper stomatal closure in response to many factors, including ABA (Negi et al., 2008; Vahisalu et al., 2008). In Arabidopsis, leaf expression of *SLAC1* is restricted mainly to guard cells (Negi et al., 2008; Vahisalu et al., 2008) and is upregulated 1 to 1.6 log2 fold change by drought (Wang et al., 2016). In contrast, we observed a strong downregulation (−2.8 to −3.6 log2 fold change) of *SLAC1* in sweet potato leaves in response to PEG (Supporting Information Figure S2; Supporting Information Table S3).

### 3.5 Varietal differences in differentially expressed genes under dehydration stress

**Figure 5:**
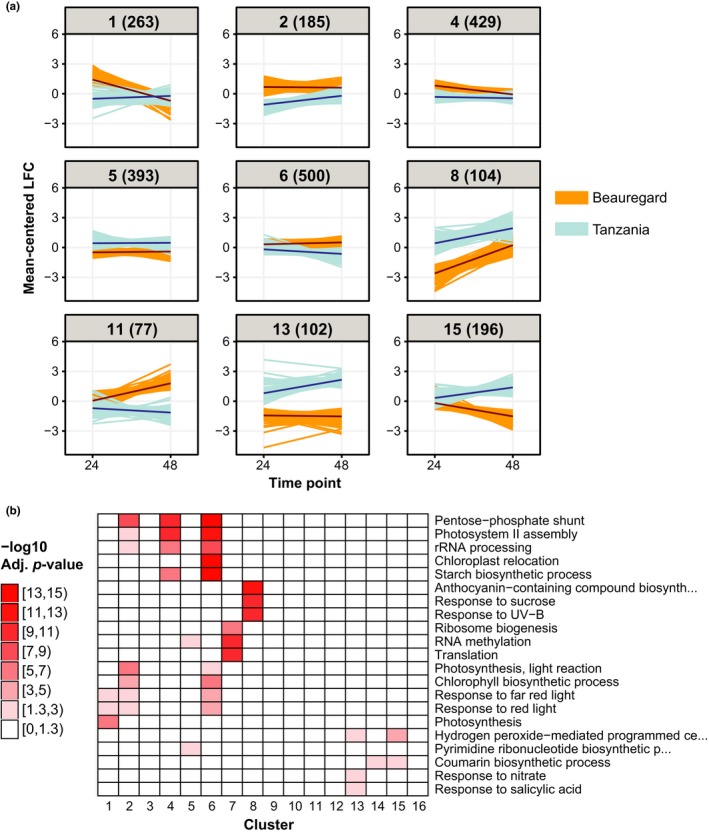
Genes differentially regulated between Beauregard and Tanzania in response to polyethylene glycol (PEG). (a) Representative K‐means clusters for mean‐centered (per gene) log2 fold changes (PEG/control) for Beauregard and Tanzania at 24 and 48 hr after stress. The number of genes in each cluster is indicated in parentheses. (b) Top three most significant biological processes gene ontology terms for each K‐means cluster

Differential gene expression analysis for each individual variety‐time point combination suggested that there are ~2,000 PEG‐responsive genes unique to each variety (Figure 2a). We hypothesize that expression differences in some of these genes contribute to the differences in days to permanent wilting point, and drought‐induced reductions in chlorophyll content and storage roots observed between the two varieties (Supporting Information Figure S1). To statistically identify genes that are regulated differentially between Beauregard and Tanzania in response to PEG, genes were tested for significant interaction effects between PEG treatment and variety, either as two‐way interactions or as three‐way interactions with time. The significant genes were classified into co‐regulated clusters using a K‐means approach in order to uncover expression patterns. In this way, 3,824 genes with significant interaction effects were identified and grouped into 16 clusters ranging from 60 to 500 genes in size (Supporting Information Figure S3; Supporting Information Table S4). Clusters 4, 5 and 6, encompassing 1,322 genes, have relatively mild differences between Beauregard and Tanzania (Figure 5a). Conversely, the other clusters, which compose the remaining 2,502 genes used for clustering, had marked differences in expression pattern between the two varieties consistent with distinct genetic responses to dehydration stress.

Additionally, these expression differences between the two varieties were dramatically different at the two time‐points in clusters 11, 13, and 15, emphasizing again that dynamic regulatory changes occur between the two time‐points. Cluster 11 was not significantly enriched for any biological process GO term, but contained an ortholog of *OST2*, which encodes an H^+^‐ATPase proton pump that is involved in ABA‐mediated stomatal closure in response to drought (Merlot et al., 2007; Figure 5b; Supporting Information Table S3). Three *OST2* orthologs were significant for interaction between treatment and variety effects (the other two were in clusters 3 and 6) and except for 24 HAS for *itf03g23220*, higher expression was observed for Beauregard for all data points suggesting that reduced *OST2* activity in Tanzania may contribute to its higher days to permanent wilting (Figure 1a, Supporting Information Figure S4). In contrast to cluster 11, clusters 13, and 15, which had increased expression in Tanzania at 48 HAS compared to 24 HAS but had either similar or weakened expression at 48 HAS compared to 24 HAS in Beauregard, were weakly significant for the biological process GO term “hydrogen peroxide‐mediated programmed cell death” (Figure 5b; Supporting Information Dataset S3). This suggests that drought‐induced hydrogen peroxide (Noctor, Veljovic‐Jovanovic, Driscoll, Novitskaya, & Foyer, 2002) causes more cell death in Tanzania. However, the overall effect of hydrogen peroxide in Tanzania could potentially not be detrimental because hydrogen peroxide is an important signal molecule for proper ABA‐mediated stomatal closure (Pei et al., 2000).

Clusters 1, 2, 4, and 6 were enriched for photosynthesis‐related GO terms and contained genes that were, in general, expressed higher in Beauregard or at comparable levels in both varieties (Figure 5). Cellular compartment GO term analysis supported an enrichment for genes involved in photosynthesis in these clusters, with significant GO terms for chloroplast thylakoid membrane, stroma and envelope (Supporting Information Figure S5a). Clusters 2, 4, and 6 were also enriched for the pentose‐phosphate shunt biological process GO term (Figure 5b). One of the products of the pentose‐phosphate shunt is NADPH (Kruger & von Schaewen, 2003), which is required for the anti‐oxidative function of glutathione reductase that may help quench reactive oxygen species (ROS) produced during drought that could damage chloroplast membranes (Das & Roychoudhury, 2014). Together, the higher expression of photosynthesis and pentose‐phosphate shunt genes in clusters 1, 2, 4, and 6 in Beauregard may contribute to its smaller reduction in chlorophyll content compared to Tanzania plants in response to drought (Supporting Information Figure S1b).

Cluster 8 genes were on average regulated at ~3 log2 fold higher in Tanzania than in Beauregard at both 24 and 48 HAS (Figure 5a). Interestingly, cluster 8 was most significant for enrichment of the GO term, “anthocyanin‐containing compound biosynthesis” (Figure 5b). Anthocyanin production is triggered by various biotic and abiotic stresses, including drought stress (Kovinich et al., 2014). While the function of stress‐induced anthocyanin is still debated, overexpression of anthocyanin biosynthesis improves drought resistance and the leading hypothesis is that it is involved in removing ROS (Gould, McKelvie, & Markham, 2002; Kovinich, Kayanja, Chanoca, Otegui, & Grotewold, 2015; Nakabayashi et al., 2014). Because ROS overaccumulation can damage not only cellular membranes but also nucleic acids and proteins, the increased expression of anthocyanin‐containing biosynthesis genes in Tanzania may contribute to its extended days to permanent wilting point compared to Beauregard (Supporting Information Figure S1a) by helping to limit levels of damaging ROS during drought stress.

## 4 CONCLUSIONS

We present here an expression profiling resource for drought stress experiments in sweet potato and have demonstrated its utility for generating hypotheses for functional genomics. Overall expression patterns were conserved between Beauregard and Tanzania, and consistent with drought responses reported in the literature, with a general upregulation of ABA signaling components and downregulation of tissue growth processes. These observations corroborate the quality of our data and give weight to the other aspects of our analysis, including several interesting findings. We have identified a group of LRR kinases that are down‐regulated at 24 HAS but not at 48 HAS suggesting their reduced activities at 24 HAS have important roles in dehydration response. Interestingly, we observed down‐regulation of single‐copy *LHCSB6* and *SLAC1* orthologs in both varieties at both time‐points. These two genes encode effector proteins, a chlorophyll‐binding component of PS II and a guard cell anion efflux protein, respectively, that have been shown to be highly important for stomatal closure during drought stress in Arabidopsis and we present these as strong candidates for overexpression experiments in sweet potato. Co‐regulated clusters of genes involved in photosynthesis and the pentose‐phosphate pathway that is expressed higher in Beauregard may contribute to its chlorophyll content being more resilient to drought than Tanzania. On the other hand, a separate co‐regulated gene cluster involved in the production of anthocyanin‐containing molecules may help increase the number of days to permanent wilting in Tanzania.

## AUTHOR CONTRIBUTIONS

C.R.B., Z.F., and M.A.K. designed the experiment. M.A.K., M.R.H, S.W., and Z.F. performed the research. K.H.L., E.C., D.C.G., and C.R.B. analyzed and interpreted the data. K.H.L. and C.R.B. wrote the manuscript. All authors read and approved the manuscript.

## Data Availability

Raw sequence reads have been deposited in the National Center for Biotechnology Information Sequence Read Archive under BioProject PRJNA475293.
