# Supplementary material for: Transcriptomic analysis of sweet potato under dehydration stress identifies candidate genes for drought tolerance
Source: Plant Direct. 2018 Oct 30;2(10):e00092. doi: 10.1002/pld3.92 (PMC6508841; doi:10.1002/pld3.92)
Supplement: Supplementary file 1 [file PLD3-2-e00092-s001.pdf]

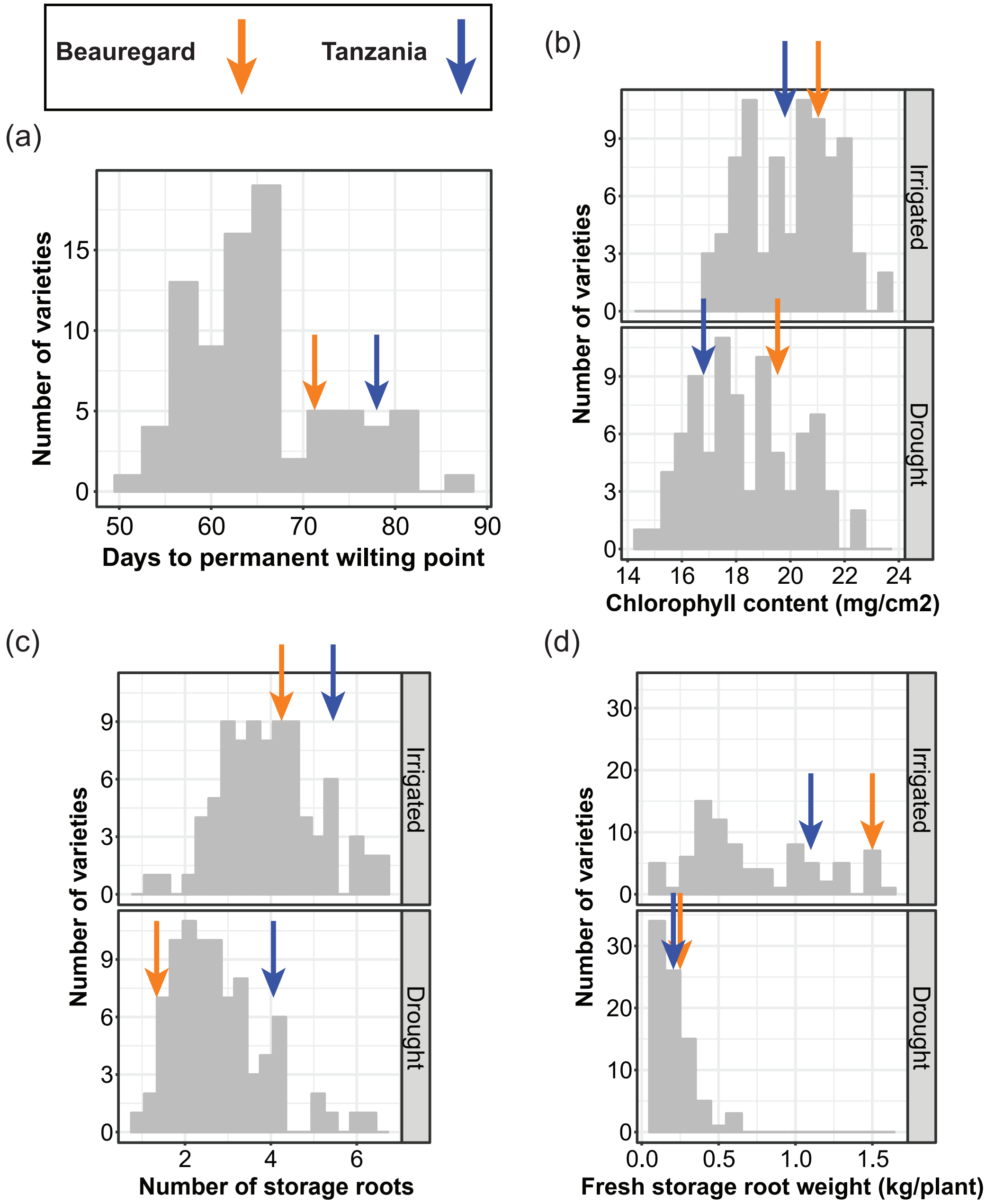

Figure S1. Phenotypic response of Beauregard and Tanzania sweetpotato varieties to drought. (a-d) Distribution of genotype means of 84 sweetpotato varieties. Data obtained from Kivuva et al. (2015) and plotted in new graphs to highlight Beauregard (orange) and Tanzania (blue). **(a)** Days to permanent wilting point (leaves and stem are dry and no longer green) recorded for plants grown under drought conditions in pots in a greenhouse. **(b)** Chlorophyll content, **(c)** number of storage roots, and **(d)** fresh storage root weight in field-grown plants under watered and drought conditions.

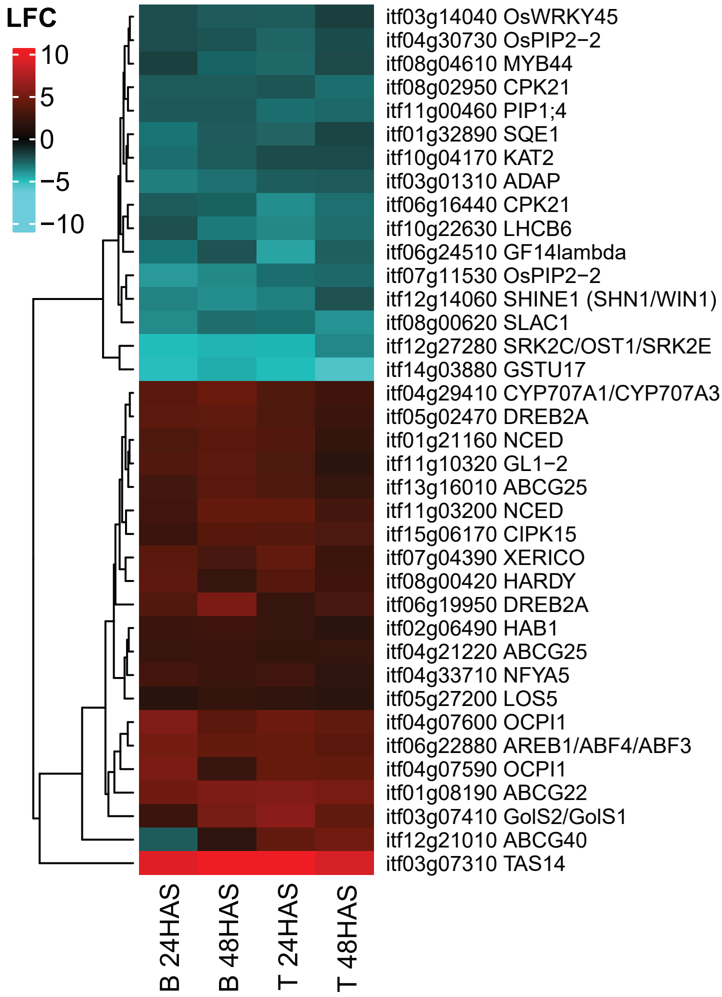

Figure S2. DroughtDB orthologs differentially expressed for all four variety-time point combinations. LFC, log2 fold changes (polyethylene glycol/control).

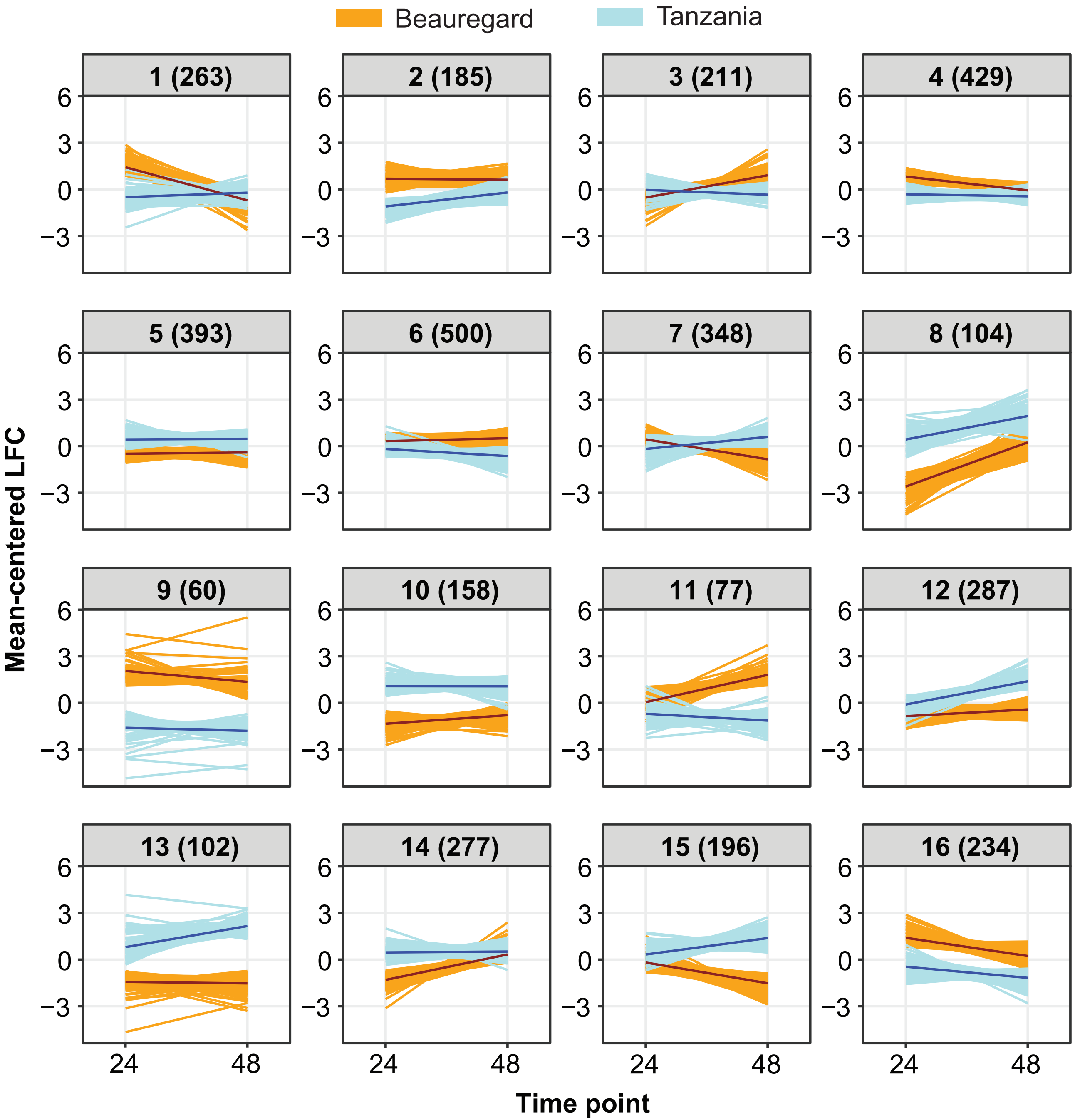

Figure S3 . All K-means clusters of genes differentially regulated between Beauregard and Tanzania in response to polyethylene glycol (PEG). The number of genes in each cluster is indicated in parentheses.

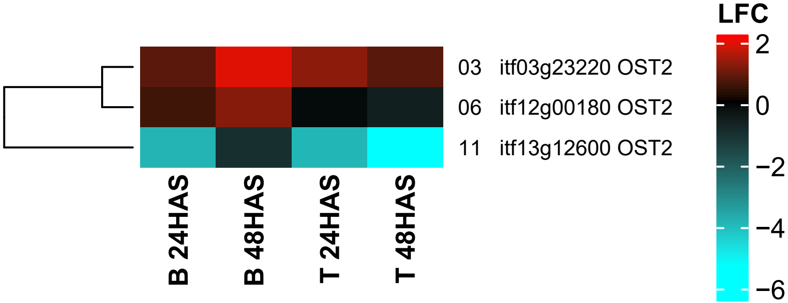

Figure S4. Expression of *OST2* orthologs that were differentially regulated between Beauregard and Tanzania in response to polyethylene glycol (PEG). Numbers to the left of the gene IDs indicate their K-means cluster. B, Beauregard; T, Tanzania; LFC, log2 fold change (polyethylene glycol/control).

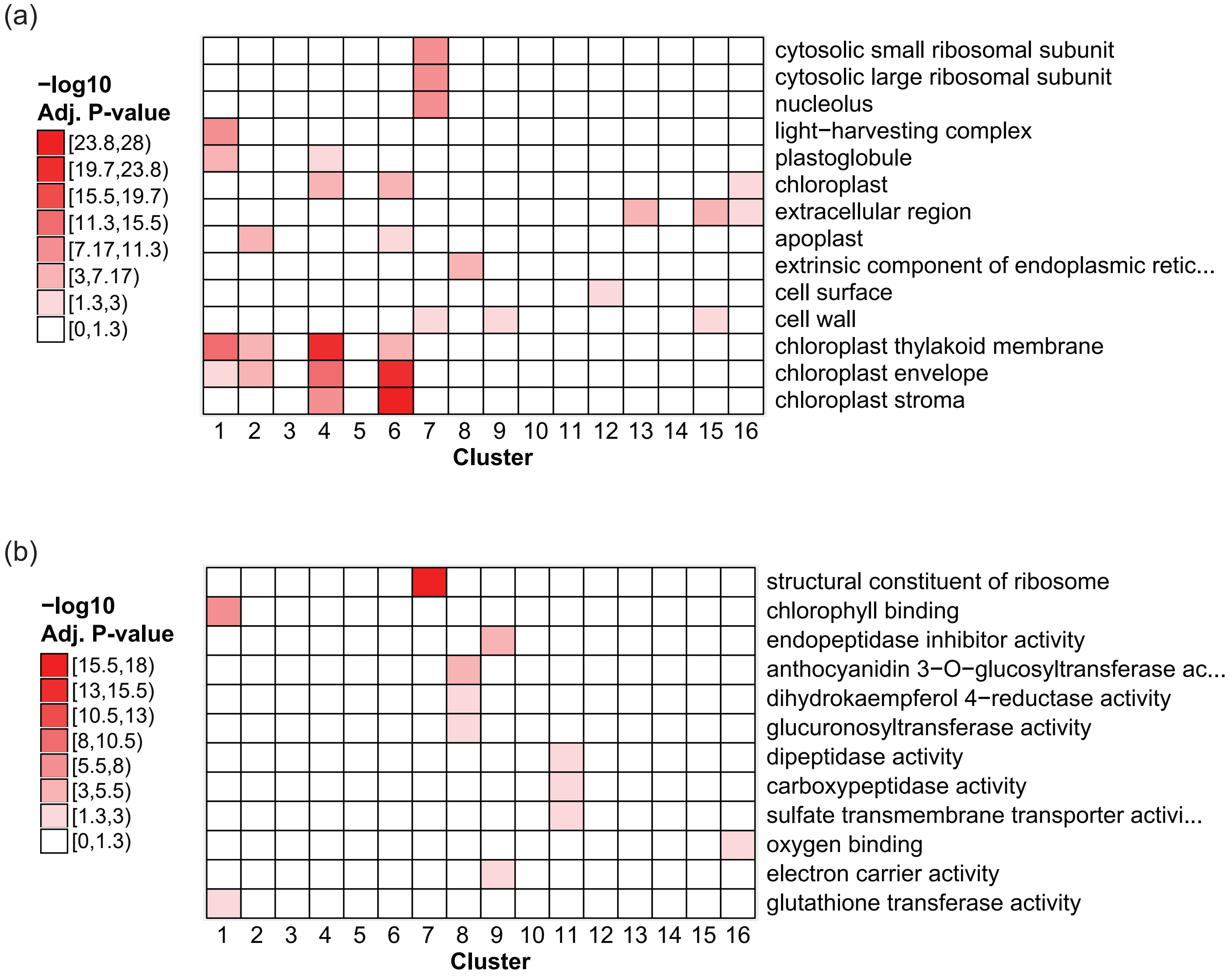

Figure S5. GO term enrichment in each K-means cluster of genes differentially regulated between Beauregard and Tanzania in response to polyethylene glycol (PEG). (a) Top three most significant cellular component GO terms for each K-means cluster. **(b)** Top three most significant molecular function GO terms for each K-means cluster.
