# Supplementary material for: Transcriptomic analysis of sweet potato under dehydration stress identifies candidate genes for drought tolerance
Source: Plant Direct. 2018 Oct 30;2(10):e00092. doi: 10.1002/pld3.92 (PMC6508841; doi:10.1002/pld3.92)
Supplement: Supplementary file 6 [file PLD3-2-e00092-s006.pdf]

Response to Reviewers' Comments
Reviewer #1:

I am a biologist and not a bioinformatician and have thus predominantly evaluated the biology behind this work rather than the validity of the computational or statistical approaches used.

This manuscript describes phenotypic characterization and transcriptomic analysis (RNA-seq) of leaves of two cultivars of sweetpotato, Beauregard and Tanzania, grown in vitro in liquid culture and treated with PEG to simulate drought conditions. Only minimal new phenotypic data are presented (low-resolution photographs of PEG-treated plantlets), but previously published data from Kivuva et al, 2015 are reproduced here in a supplemental figure that plots "days to wilting" and other characteristics (chlorophyll levels, number of storage roots and fresh root weight) under optimal and drought conditions for multiple cultivars including Beauregard and Tanzania. For the RNA-seq data, lists of PEG-induced/repressed genes were run through Gene Onthology, and most functional categories do make biological sense (e.g., response to water deprivation and to abscisic acid; or cell proliferation and response to auxin among downregulated genes). No RNA-seq data validation by other approaches (e.g., by qRT-PCR) nor functional characterization of differentially expressed genes (e.g., overexpression or knockout in a model organism) were performed.

For well-studied drought-related genes from model organisms, in the RNA-seq experiment performed herein, differential expression of putative sweetpotato orthologues was observed for 122 out of 300 genes, and the authors discuss the potential role of some of those genes in drought response of sweetpotato. The authors also speculate that PEG-mediated repression of other genes (known to improve drought tolerance when overexpressed, such as LHCB6) may be the reason for the reduced plant performance under drought and suggest that increasing expression of such genes (e.g., via transgenic overexpression) may help to optimize plant yield under drought.

Finally, K-means clustering was performed in this manuscript on genes differentially regulated between Beauregard and Tanzania. Some of the clusters are enriched for biological processes that may be at least in part responsible for the differences in the response of the two cultivars to drought, but again, no functional validation of those assertions is provided. The authors argue that their analysis provides a list of candidate loci for future efforts towards improved drought tolerance. However, given that (1) all of these genes are categorized as potential targets for transgenic manipulation (or breeding efforts) based on prior work in model organisms (rather than current work, as no new candidates are being considered) and that (2) no functional confirmation was carried out for sweetpotato genes to suggest the ability of any of the candidates to alter drought responses of this species, I am not sure how/why we are better off now at prioritizing the existing 300 candidates than we were before this study. I would think that irrespective of a

gene being or not being differentially expressed in sweetpotato under drought (or even irrespective of whether it is transcribed or present in the genome of this species), transgenic overexpression of the candidate gene (let's say identified as improving drought tolerance in Arabidopsis) may lead to better sweetpotato survival/yield under drought.

Response: We agree with reviewer #1 that confirming the roles of specific genes will require molecular approaches such as making transgenic plants. However, this approach would be time-consuming and outside the scope of this study which was discovery of candidate genes involved in drought stress that can be integrated with future QTL studies on drought in sweetpotato. Thus, the work presented here will help to prioritize candidate genes to be explored in future experiments, including an ongoing QTL project within our group.

Furthermore, although orthologs shared across species often have similar functions, this is not always the case and additional evidence such as expression data helps identify the most promising candidate genes. For example, as you mentioned, only 122 of 300 putative orthologs of known drought response genes responded to dehydration stress in cultivated sweetpotato in our dataset. Additionally, this dataset will help corroborate novel candidate genes, not included among these 300 orthologs, that may be identified in sweetpotato in the future, for example by using the Beauregard x Tanzania mapping population as discussed in the manuscript. In this regard, our analysis of varietal differences between Beauregard and Tanzania will be especially informative.

How does PEG response in young plantlets grown in tissue culture relate to the drought response of mature plants grown in the field? In that regard, it would have been helpful to know what percentage of the plantlet-expressed drought-related genes identified are also transcribed in field-grown adults (and a qRT-PCR validation of candidate plantlet genes would have been an appropriate approach here).

Response: We do not have enough information to say “what percentage” of overlap there is between drought/dehydration responses in PEG-treated plantlets grown *in vitro* versus plants in the field. Clearly, there will be some differences, but we also expect there to be substantial overlap and this idea is supported by the observation that many drought-related genes identified previously in the literature were responsive in our PEG treatment data.

We feel that an *in vitro* PEG treatment approach allowed us to achieve a level of experimental consistency, as evidenced by the tight clustering of our biological replicates (Figure 1b in the manuscript), that would have been difficult to attain if we had imposed actual drought in a field or greenhouse setting. Field evaluations are particularly challenging because drought effects can be confounded by other stresses such as heat or pests, and these effects can be very different in different parts of the field. For this reason, field experiments are often performed in multiple locations, multiple years with many replicates, but this type of experimental design would be very costly for an RNA-Seq experiment, which

is what we would really need to answer the “what percentage” of overlap question. As stated above, we agree that more work will be needed to confirm the roles of specific genes, but we believe that our work presented here will be a valuable resource for prioritizing candidate genes for such work.

Also, how do the results of this study compare to that reported in other drought-related manuscripts mentioned in lines 89-97 (i.e., what is the overlap)? What tissues were analyzed in these other studies?

Response: It is difficult to assess the amount of overlap between our data and these other datasets because their expression values were calculated by aligning reads to their own transcriptome assemblies and unfortunately, the transcriptomes in these studies are not publicly-available. Furthermore, results from such comparisons may be challenging to interpret. Data from Solis et al. 2016 and Peng et al. 2017 were un-replicated and involved species other than *I. batatas*. Data in Yang et al. 2018 were duplicated, but examined a different *I. batatas* cultivar and used whole-plant RNA instead of leaves specifically as in this study.

No RNA-seq library summary is provided in the Materials and Methods. For example, how many reads per replicate per library were obtained and how many genes were detected as expressed? I could not easily extract that data from the supplemental Excel files provided and I am not always able to interpret what the gene lists in supplemental datasets are. Please, annotate supplemental files (what are, for example, DP, CC and MF?).

Response: A new Supplemental Table has been added to describe the number of reads for each library. FPKM values are available for each library in the Table S1 to assess gene expression. Titles describing the contents of the supplemental datasets are available in the ‘Supplemental Materials’ section of the ‘Materials and Methods’ and these have been edited to include definitions for BP, CC and MF, which stand for the different categories of GO terms: biological process, cellular compartment and molecular function.

While I understand that Beauregard and Tanzania are representative US elite and African landrace varieties, they are not that different in their drought response in terms of total yield (despite some differences in chlorophyll levels and storage root number). Why were these specific cultivars chosen? I would think that comparing a drought tolerant and sensitive cultivars from the same geographic region could have been more informative than looking at genetically very diverse cultivars with similar yield under drought. Even without someone having performed this study, I would have expected to see some transcripts in common and some unique among differentially expressed genes in any two cultivars.

Response: While it is true that a comparison between strongly drought-tolerant and drought-sensitive genotypes would be another interesting RNA-Seq experiment, our study provides expression data specifically for two agriculturally-important sweetpotato varieties. Experiments using other varieties may shed light on some aspects of drought response in sweetpotato but may not be representative of expression patterns in widely-used cultivars. Furthermore, we are in the process of identifying QTL for drought-related traits using a Beauregard x Tanzania mapping population and the expression data presented here will help identify candidate genes from these QTL.

Other concerns I have regarding this work are listed below.

Lines 82 - 84, the plantlets stay greener under what conditions? When not watered? Specify.

Response: The sentence has been modified to specify that this was under drought conditions.

Lines 89-90, why do the authors state that prior work did not look at drought tolerance of cultivated I. batatas, if later in the same paragraph they cite two such studies (Yang et al. and Solis et al.)?

Response: We have edited these sentences to describe the status of current literature on global transcriptome response in *Ipomoea* species more clearly. To avoid confusion, we have removed the sentence referencing Solis et al. 2014, which reported only qRT-PCR results for a small number of genes and is not an example of a global transcriptome study.

Lines 97-99, it's important for the authors to share some information about the genetics and the evolutionary history of sweetpotato and state the currently available reference genomes of Ipomoea species before stating why RNA-Seq reads from I. batatas were aligned against the I. trifida genome.

Response: We have added text in the introduction to explain our choice for the reference genome. See also our response to comment #2 by reviewer #2.

Lines 111-116, the description of the MBP culture medium lists autoclaving after the addition of growth regulators, which I assume is a mistake (as these are usually heat sensitive). It would have been helpful if those supplements that are added after autoclaving were stated as such.

Response: The MBP medium used in the current study is recommended to be autoclaved by the supplier. We have clarified this in the text.

Lines 118-121, if I understand this correctly, different amounts of PEG were added to MBP to make PEG-containing media of different osmolarity. If so, would not that change the volume and, accordingly, the MBP strength, with the final concentration of MBP lower than 1x and different in different PEG concentrations? I find this odd and would think that MPB should have been kept at 1x for fair comparisons, irrespective of the concentration of PEG (for example, by starting with 2xMBP, powder PEG and water to bring MBP to 1x after the addition of PEG).

Response: We used liquid MBP media for the experiment and therefore the PEG was dissolved in the media without affecting volumes, just the concentration. This has been clarified in the text.

Lines 119-123, how where the osmotic pressure values deduced or measured?

Response: These were estimated from previously published osmotic pressure values for various concentrations of PEG 6000. This has been clarified in the text.

Lines 129-130, were the nodes fully submerged into MBP?

Response: Only the internode preceding the node was submerged in the media. The node was left out of the media to allow growth.

Lines 131-133, from the description provided, I am unable to visualize the plastic that holds the plants. If a non-standard setup is used, inclusion of a supplemental figure would have been helpful.

Response: It is difficult to see the acrylic plastic used in this study because it is as colorless as the containers used in the experiment. The purpose of this acrylic plate was to make sure that only the roots are in contact with the liquid media. Please see Figure 1 below for the setup.

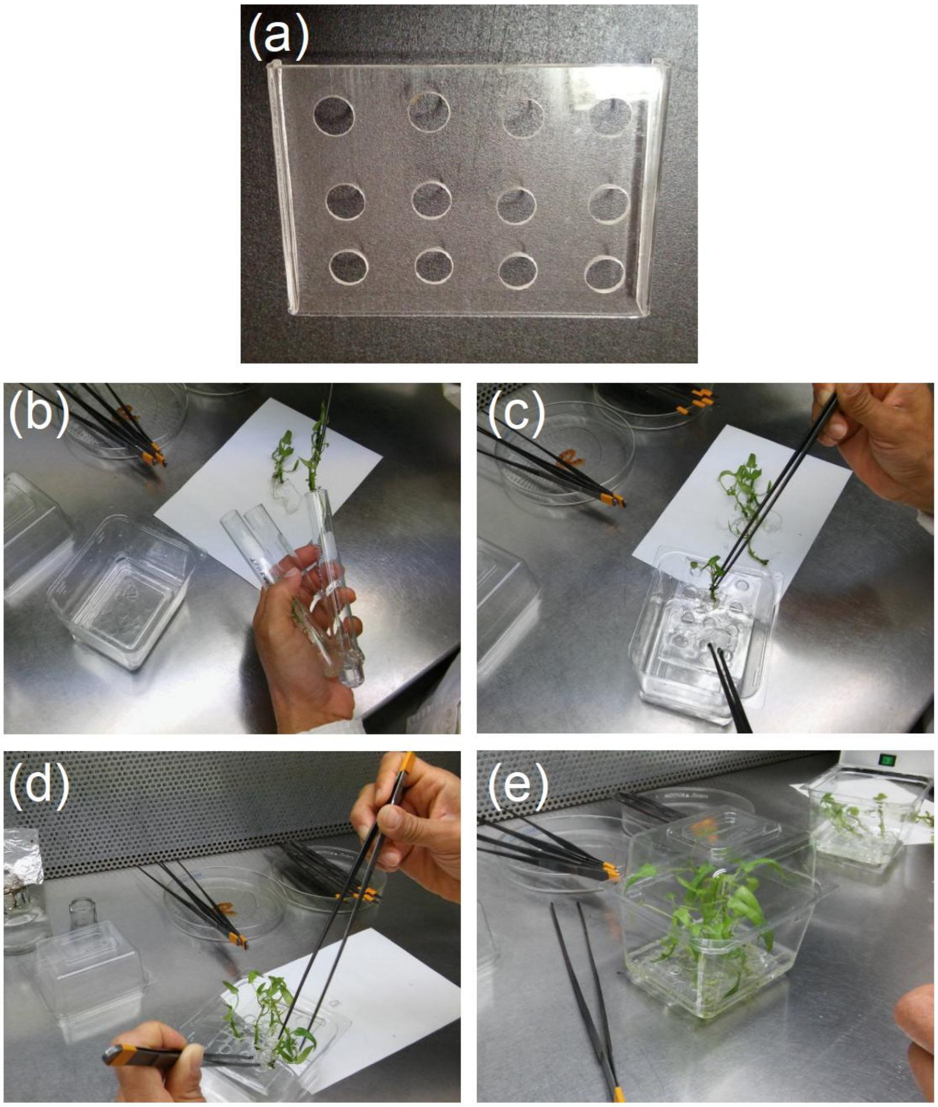

Figure 1. Procedure for transferring plantlets to liquid MDP media. (a) Acrylic plate for preventing aerial parts of the plant from touching the media. (b) Removal of plantlet from solid media used for the initial 14 days of growth from cut node. (c) Fitting a plant through one of the holes in the acrylic plate. (d) Fitting additional plants through other holes in the acrylic plate. (e) Final setup for growth in liquid MDP media.

Lines 405-413, I found this argument confusing. By saying that the genes "lack paralogs", I assume the authors mean that these are single-copy genes in I. trifida (as shown in fig 4C). What I do not understand is what makes the authors believe that in I. trifida SLAC1, LHCB6 and KAT2 are also down-regulated by drought. Where does the expectation (that a hexaploid species I. batatas and it's diploid ancestral species I. trifida should behave the same with respect to these genes' expression under drought) come from? Even between the two cultivars of the same species many genes have the opposite expression patterns. From the transcriptomics data of the authors, can it be inferred how many SLAC1-, LHCB6- and KAT2-like genes (alleles, homeologs or homologs) there are in the genome of I. batatas?

Response: Actually, we were referring to the regulation of these genes in cultivated sweetpotato, *I. batatas*, not in *I. trifida*. The single-copy nature of these genes described in the paper refer to the *I. trifida* genome and while it is possible these genes could exist in more than one copy in the cultivated sweetpotato genome, reads from those paralogs should map to the same target gene in the *I. trifida* reference genome. Thus, the interpretation that these genes (potentially across paralogs) are down-regulated in Beauregard and Tanzania under dehydration stress should be valid regardless of whether these genes are single-copy in *I. batatas*.

Reviewer #2:

1. I think this work is very meaningful. The analysis is detailed, and the results support the conclusion.
2. About the methods, line 154 and 177.
Why use the I.trifida genome assembly V3 and not use the sweetpotato reference genome (Yang et al. 2017)?
Yang J, Moeinzadeh MH, Kuhl H, Helmuth J, Xiao P, Haas S, Liu G, Zheng J, Sun Z, Fan W, Deng G, Wang H, Hu F, Zhao S, Fernie AR, Boerno S, Timmermann B, Zhang P, Vingron M (2017) Haplotype-resolved sweet potato genome traces back its hexaploidization history. Nature Plants. doi:10.1038/s41477-017-0002-z

Response: We have been analyzing the hexaploid genome sequence reported in Yang et al. and have multiple lines of evidence that indicate the genome assembly is of poor quality; these results are included in a separate manuscript that is currently under review. In addition to likely mis-assemblies indicated by inconsistencies with mate-pair reads and alignments with BAC sequences, we found that the assembly presented in Yang et al. contains only 73.3% full-length BUSCOs, indicating that many genes would be missed if this assembly was used as a reference genome for our studies.

3. Some format need to be checking, such as the following:
Line 99, an I. trifida reference genome, which one? Should be citing.
Line 93, I. trifida was the hexaploid I.trifida, line 99 I. trifida was the diploid I. trifida. Different ploidy should be distinguished.

Response: These sentences have been edited to clarify these points.

Line 111, CIPNUMBER◊ CIP NUMBER or CIP Number

Response: This has been corrected in the text.

Line 681.

Response: We are not sure what the reviewer was referring to (there was no comment here besides the line number), but we noticed that some of the authors on this citation had a superscripted number beside their names because of a glitch with the citation manager. This has been fixed.
